# Improving understanding of clinical trial procedures among low literacy populations: an intervention within a microbicide trial in Malawi

**DOI:** 10.1186/1472-6939-13-29

**Published:** 2012-11-08

**Authors:** Paul M Ndebele, Douglas Wassenaar, Esther Munalula, Francis Masiye

**Affiliations:** 1 HJF-DAIDS, NIAID, NIH, 6700A Rockledge Drive Room 42A193, Second Floor, Bethesda, MD, 20817, USA; 2 University of KwaZulu Natal, Pietermaritzburg, South Africa; 3 University of Zambia, Lusaka, Zambia; 4 College of Medicine, University of Malawi, Blantyre, Malawi

**Keywords:** Assessment, Comprehension, Double-blinding, Informed consent, Intervention, Randomisation, Placebo, Malawi, Understanding

## Abstract

**Background:**

The intervention reported in this paper was a follow up to an empirical study conducted in Malawi with the aim of assessing trial participants’ understanding of randomisation, double-blinding and placebo use. In the empirical study, the majority of respondents (61.1%; *n=*124) obtained low scores (lower than 75%) on understanding of all three concepts under study. Based on these findings, an intervention based on a narrative which included all three concepts and their personal implications was designed. The narrative used daily examples from the field of Agriculture because Malawi has an agro-based economy.

**Methods:**

The intervention was tested using a sample of 36 women who had been identified as low scorers during the empirical study. The 36 low scorers were randomly assigned to control (n=18) and intervention arms (*n*=18). The control arm went through a session in which they were provided with standard informed consent information for the microbicide trial. The intervention arm went through a session in which they were provided with a narrative in ChiChewa, the local language, with the assistance of a power point presentation which included pictures as well as discussions on justification and personal implications of the concepts under study.

**Results:**

The findings on the efficacy of the intervention suggest that the 3 scientific concepts and their personal implications can be understood by low literacy populations using simple language and everyday local examples. The findings also suggest that the intervention positively impacted on understanding of trial procedures under study, as 13 of the 18 women in the intervention arm, obtained high scores (above 75%) during the post intervention assessment and none of the 18 in the control arm obtained a high score. Using Fischer’s exact test, it was confirmed that the effect of the intervention on understanding of the three procedures was statistically significant (p=0.0001).

**Conclusions:**

Potential trial participants can be assisted to understand key clinical trial procedures, their justification and personal implications by using innovative tailored local narratives.

## Introduction

Several authors have reported or discussed some difficulties that research participants face in understanding clinical trial concepts. Various studies have also confirmed that trial participants have problems understanding the differences between research and routine care [1-8]. There is general agreement that the trial concepts of randomization, double blinding and placebo are difficult to explain to study participants since they are part of the scientific language that low literacy populations are not familiar with [3-8]. Limited understanding of trial procedures suggests that what is often termed informed consent may not be adequately informed and goes against the principle of respect for persons which requires that individuals understand the information they are provided with before making decisions on participating in a trial. Limited understanding may also be a sign that the kind of information being received by trial participants, as well as the methods used in providing the information to study participants, may not be optimal.

In order to try and address the problem of limited understanding, various studies have been conducted globally to test interventions aimed at improving trial participants’ understanding of research participation, including clinical trial procedures. Some studies have attempted to introduce flexibility in the process of information disclosure in order to suit the information needs of the potential trial participants [9-11]. Some have considered supplementing informed consent information using video or computer technology [12-14]. In contrast, others have looked at the usefulness of educational booklets as a way of supplementing information while other studies have considered the provision of information in a modular and hierarchic approach [14-16]. Other studies have sought to assess the usefulness of trial specific tailored information while others have focused on reducing the reading levels of informed consent documents. Some have considered the use of simplified informed consent forms [17-20]. Realizing that children also take part in trials and have unique information requirements, one specific study has looked at the development of informed consent documents for children [21]. In recognition of the fact that much research is conducted among low literacy populations globally, some studies have tested interventions aimed specifically at addressing information disclosure in low literacy populations [22-24]. One study tested the use of a decision aid kit in informed consent while another tested an intervention aimed at improving understanding of placebo use [25,26].

Reported interventions have focused on different areas of emphasis ranging from modifying the informed consent process, supplementing the informed consent information to simplifying the information through lowering reading level or simplification of the informed consent forms. Review of reports on interventions confirms that some of the more effective interventions were based on the following characteristics: better organized processes, shorter and more readable informed consent documents, simplified and illustrated formats, and corrected feedback [27-29]. The reviewers point out the challenge of distinguishing between understanding and recall in some of the studies and suggest the use of various interactive techniques in efforts to improve understanding. These reviews concluded that efforts to improve understanding through the use of multimedia and enhanced informed consent forms have had only limited success. The limited success may be attributed to the fact that the so called video or computer programmes may just be a repetition of the same information that is provided through the informed consent form without much change.

A critical review of previous interventions revealed various weaknesses which could have reduced the impact of some of the interventions. By looking critically at the information that was disclosed through some interventions, it was evident that some interventions assumed that people know what research entails [14]. It was clear that some interventions assume that people are familiar with clinical trial procedures [9]. Some interventions did not assist in making individuals aware of the trial procedures and their purposes [17]. It was also evident that some interventions did not deal adequately with the personal implications of research participation and of specific trial procedures [17]. These observations are supported by the review of interventions which concluded that some of the interventions that are developed to improve understanding are poorly thought out and are merely a repetition of the information from informed consent documents using different media [28].

The intervention reported in this paper was developed in response to findings from an empirical study (under review) that identified some respondents who scored low (<75%) on measures of key elements of understanding of clinical trials. The intervention had been anticipated even before initiating the study because several studies conducted elsewhere found low levels of understanding among study participants [3-8]. It would have been unethical to ignore this problem after identifying it empirically. The researchers were also convinced by available literature suggesting the efficacy of well thought out interventions [10,21,22,30].

### Objectives of the intervention study

The intervention study was based on the premise that potential trial participants can understand trial procedures and their personal implications if the explanations that are provided to potential participants about the procedures include details on how the procedure will be implemented, and the justification and personal implications of those procedures. The primary aim of the intervention study was therefore to design, implement and test the usefulness of a pilot intervention aimed at improving understanding of randomisation, double-blinding and placebo use and their personal implications.

## Methods

The intervention designed as part of this study was based on the nature of data yielded by our empirical study (under review) conducted in Lilongwe and Blantyre at two sites of a multi-country microbicide trial between 2008 and 2009. The empirical assessment study assessed trial participants’ understanding of randomisation, double-blinding and placebo use and their personal implications. In the empirical study, the majority of respondents (61.1%; *n=*124) obtained low scores (lower than 75%) on understanding of all three concepts under study. The intervention reported in this paper used everyday examples in explaining clinical trial procedures and their implications. African cultures are generally well known for story telling as a way of educating individuals [31,32]. Stories with some meaning are often told as a way of ensuring that individuals understand a particular issue. The intervention did not make any assumptions about pre-existing knowledge and it was based on the review of existing interventions and studies aimed at testing some related interventions [9,11,12,14-17,19,20,29].

The intervention was mainly based on Faden and Beauchamp’s psychosocial schema, which views informed consent as being made up of three sequential behavioural steps: (a) reception, (b) comprehension and (c) utilisation of the comprehended information in reaching a decision whether or not to participate in a study [33]. The schema postulates that for consent to be informed, a prospective trial participant has to go through the three steps. The intervention also borrowed from the Meerwein model of information processing [34]. Meerwein's model defines three main dimensions of the informing process, namely (a) the information itself, (b) the emotional dimension concerned with rapport between the researcher and the participant, and (c) the interactional dimension which is concerned with the capacity and willingness of the research staff to perceive and discuss emotional needs, concerns and complaints of trial participants and to deal with these.

The main components of the intervention consisted of a PowerPoint presentation which included a mix of the following approaches:

•A hierarchical and modular approach to providing information. This entails providing information in manageable sections. The information becomes more complex as the presentation proceeds [16].

•Use of vignettes in explaining the trial concepts and research [35].

•Colourful pictures were included in the presentation to supplement written information and the discussions about microbicides and the trial [9,12,24]. Purpose, justification and implications of research participation and trial procedures were also included [4].

•Asking patients to repeat in their own words or explain to others [35].

•Use of other appropriate ways of ensuring personal understanding, including inviting research participants to discuss with other participants [36,37].

•Use of a neutral team of intervention staff distinct from the research team in group discussions with trial participants. These were persons who had been trained to teach potential participants about the key methodologic aspects of research and who had experience in research [38].

**Figure 1:**
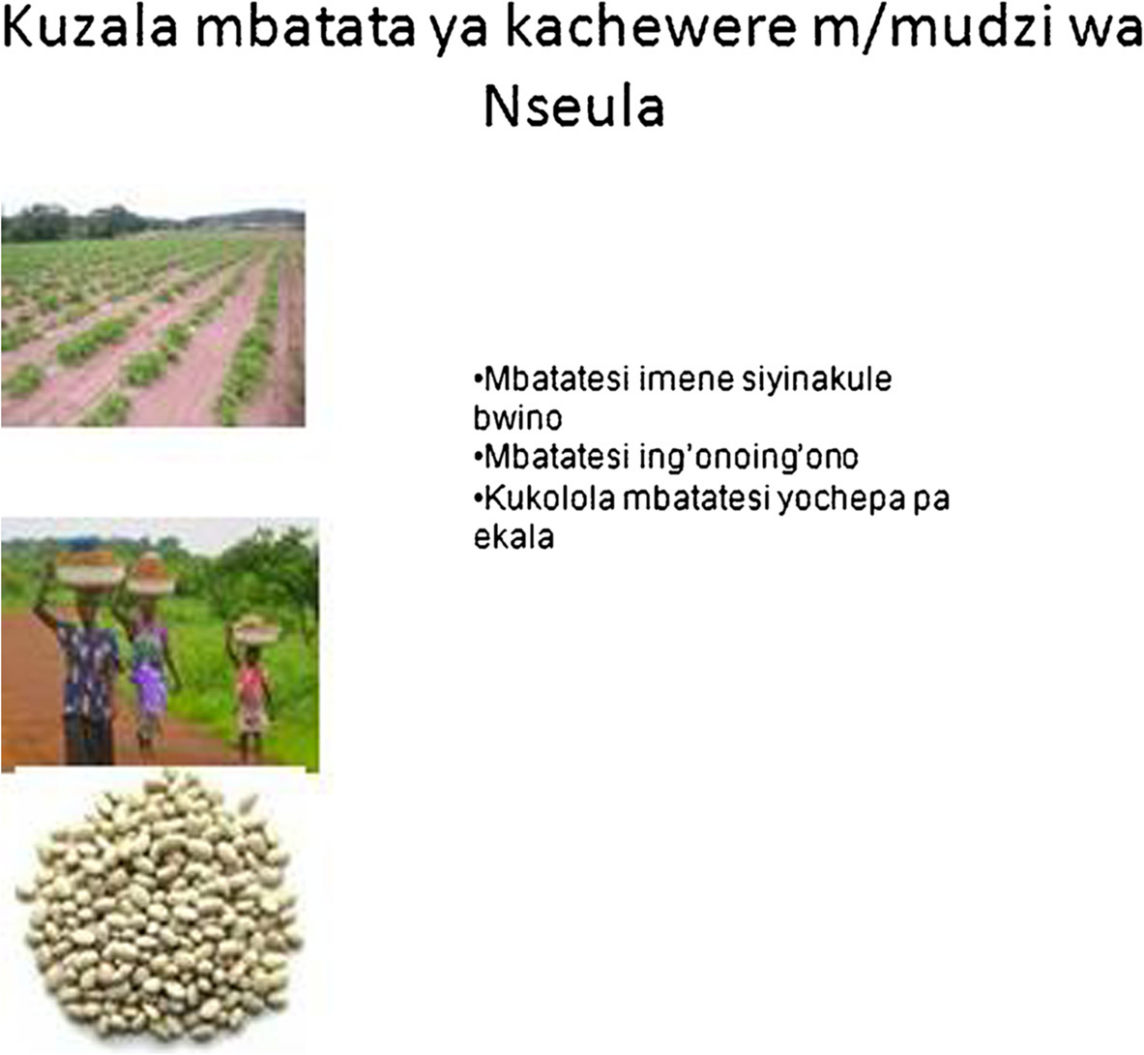
Slide showing the problem of low potato yields in Ntcheu area.

**Figure 2:**
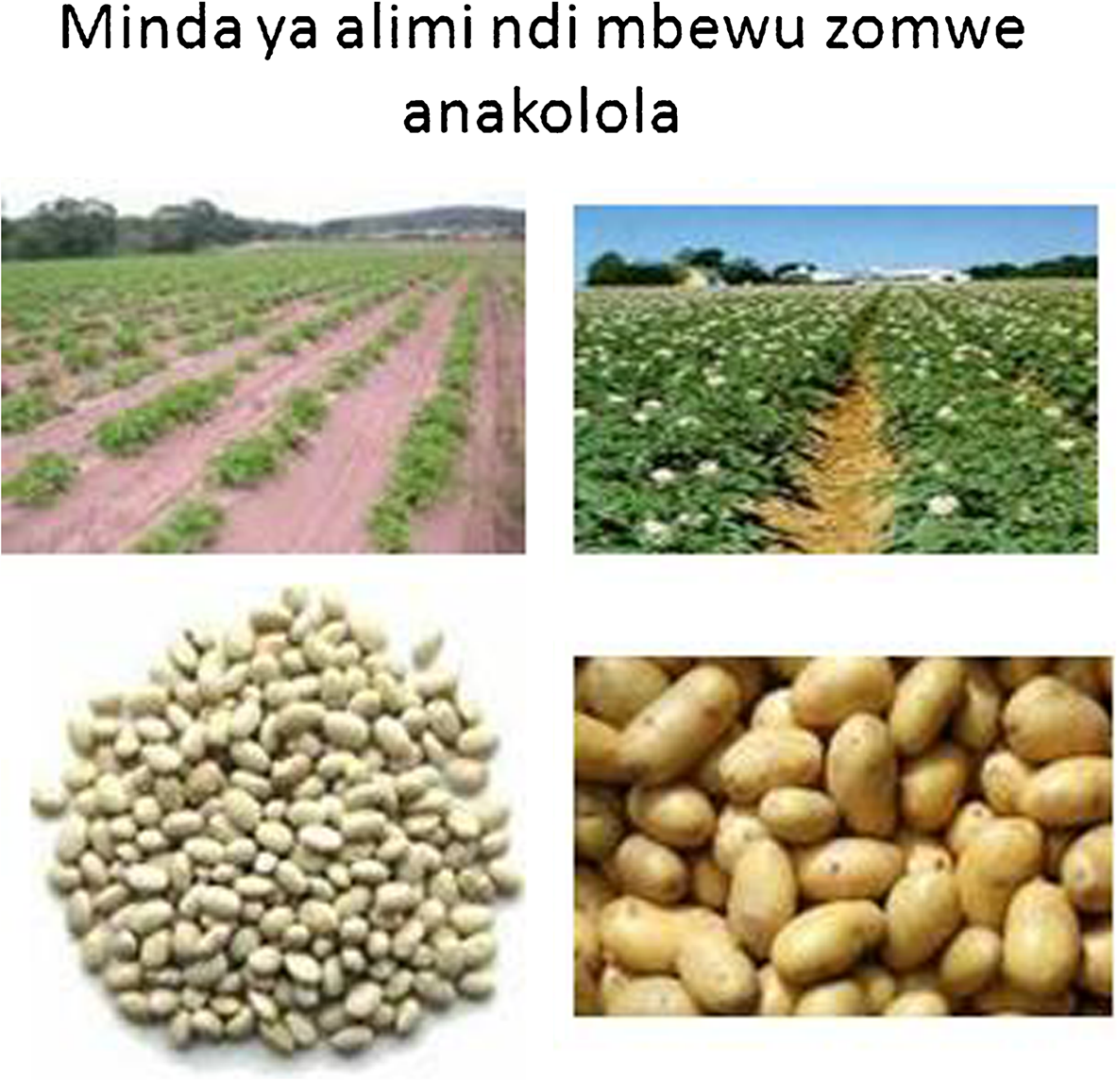
Slide showing results from different potato plots.

**Figure 3:**
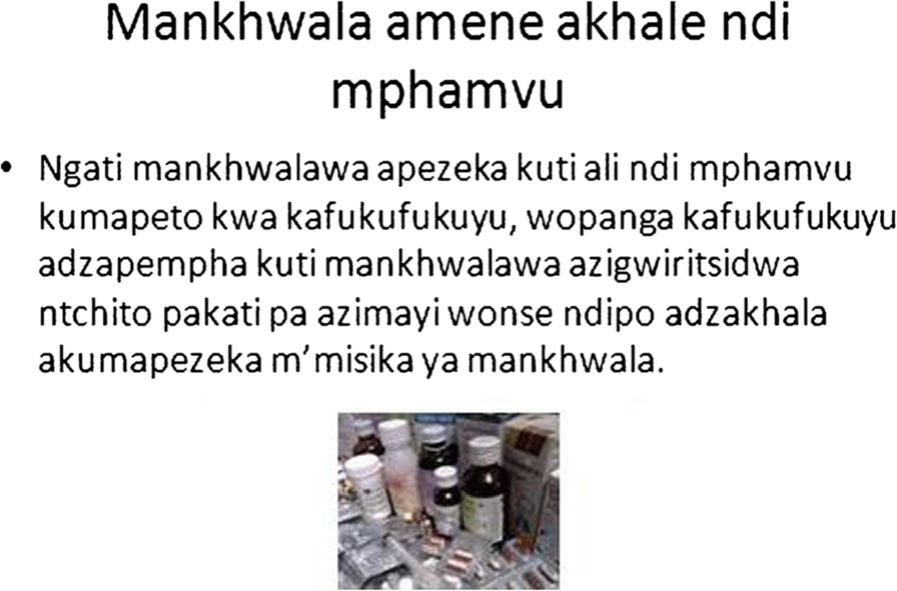
Slide linking clinical trials to the medicines that are available in pharmacies, hospitals and stores.

The intervention was implemented in the form of a narrative which was given in ChiChewa, the local language, with the assistance of a PowerPoint presentation. Figures 1, 2, 3 below show some of the slides that were used in the intervention. The intervention was based on a story about a company which intended to test a new fertilizer in an area (Ntcheu) where farmers were experiencing very low potato yields. Ntcheu is well known throughout Malawi for Irish potato production. Using the fertilizer story, the concepts of research, randomisation, double-blinding and placebo use were illustrated including the reasons why research is necessary and why these procedures were employed as well as the personal implications of these procedures to the farmers in Ntcheu. In the narrative, the farmers were given some eligibility criteria (including having one acre plot and willingness to participate). The farmers were randomised by picking small pieces of paper from a hat that were numbered from 1–100. These numbers would determine the “fertiliser bag” that each farmer would take home. There were 100 bags all of the same colour and 50 of them contained the test product (the fertiliser) while 50 contained “some material” which looked exactly like the test fertiliser but did not have any of the chemicals in the test fertiliser (placebo). The farmers and the agriculture extension workers were both not aware of which study arms farmers had been assigned to since the test product and the placebo had been packed in bags which looked similar. The intervention covered the application of the procedures as well as the interpretation of the findings from the fertiliser study. This was aimed at ensuring that the intervention promoted a fuller understanding of research and trial procedures. After narrating the story, the presenter then related the Irish potato fertiliser research narrative and the procedures of the microbicide clinical trial, including the trial concepts under study (as well as their justification and personal implications).

The intervention was implemented on 20^th^ August 2009 at the Blantyre site only for logistical and budgetary reasons. All follow-up activities of the microbicide trial had already come to an end and participants had been informed about the findings from the microbicide study in March 2009. This therefore meant that the intervention did not in any way impact on the microbicide study since the microbicide study activities had already come to an end. The microbicide study had established that the two products which were being tested were not effective in protecting women against HIV infection [39]. The intervention was approved by the principal investigator at the Blantyre Site after being briefed about the findings from the empirical study that had tested understanding of the three trial concepts. The principal investigator then informed the study team members about the impending intervention and requested them to support the intervention by providing space and logistical support.

A list of the low scorers from the Blantyre site was provided to the microbicide study staff so that they could assist with the tracing of the women who had participated in the empirical study. From a list of 77 participants who obtained low scores at the Blantyre site, current contact details could only be found for 63 participants who were still based in Blantyre. It was noted that 56 of the 63 participants who were identified were based in Manyowe and Manase areas. A decision was therefore made to follow-up only the 56 participants from Manyowe and Manase areas. Staff who were employed as field tracers at the microbicide trial site were requested to visit the homes of all 56 participants. It is important to note that during the empirical study, the women had given permission to be re-contacted for the purpose of continuing with the intervention. The researcher in the current study offered transport as well as other logistical support to the field tracers. The field tracers found 38 women at their homes and invited them to visit the study site for the intervention study. For the remaining 18 who were not available, the field tracers left messages inviting them to visit the study site at 8:00am on Thursday August 20, 2009.

On Thursday 20^th^ August 2009, by 8:30am there were 39 women present and a decision was made to precede with the study activities. All 39 women had scored less than 75% during the initial assessment. Informed consent was sought from all 39 women after disclosure of information by a study team member. The information which was provided included reminding them about the microbicide study, the empirical study on understanding of procedures, and then requesting their consent for the intervention study. Three women indicated that they could not spend more than one hour at the site as they had to collect their children from school and were accordingly excluded from the study activities and reimbursed for transport expenses. Three women arrived more than 25 minutes after the two sessions had already begun. The three were not invited to join as they would have affected the flow of activities. They were, however, offered some refreshments and reimbursement for transport, and were given the opportunity to meet the microbicide study nurses for any issues that they might want to discuss with them.

The remaining 36 women indicated verbally that they were consenting to continue with their participation in the study and were prepared to go through all the remaining activities of the current study (Note that at this time, the microbicide study had already been terminated). The 36 women were accordingly randomised into two groups using small papers numbered from 1–36. All those who picked odd numbers were assigned to the intervention arm and those who picked even numbers were assigned to the non-intervention arm which was going to receive standard microbicide trial informed consent information.

A trial nurse responsible for obtaining informed consent was requested to present standard informed consent information on the microbicide study to the 18 women in the non-intervention group, in addition to health information on cervical cancer, and was advised to allow the women to ask questions. Our ethical concern here was to make the non-intervention group ‘more than placebo’ by imparting some useful women’s health information unrelated to the information presented in the intervention arm.

**Figure 4:**
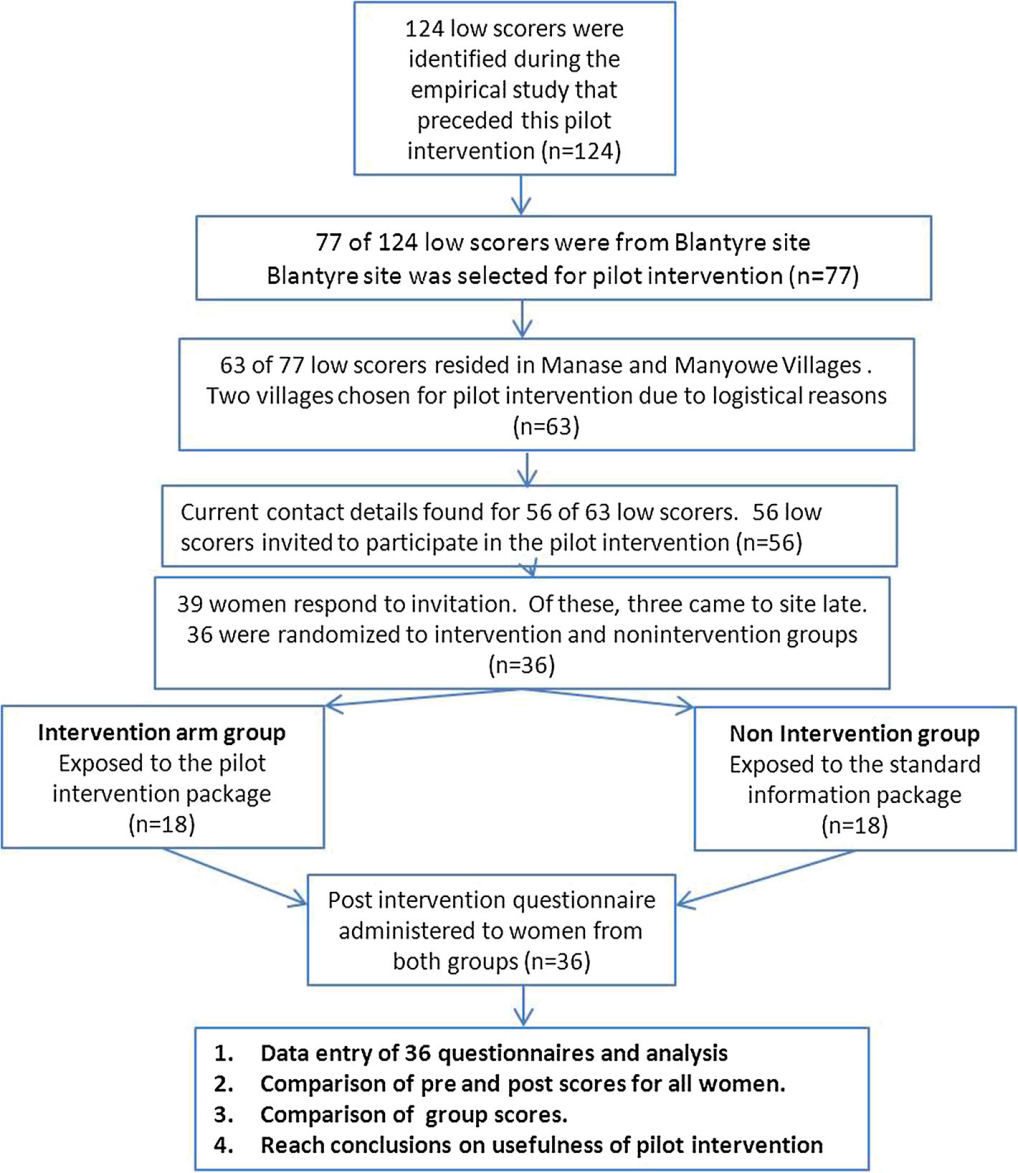
Illustration of pilot intervention procedures.

The two sessions began at the same time and the non-intervention arm session ended about 30 minutes earlier than the intervention session. The women were then invited for individual structured interviews on a one on one basis. There were two research assistants administering the questionnaire and the two groups were kept in separate rooms and there was one study team member who coordinated the movement of the women from the two sessions to the separate rooms that had been assigned for the post-intervention interviews. Each interview took on average 15 to 20 minutes since the post-intervention questionnaire was shorter than the initial one (it only included questions on the 3 concepts as well as their implications). Figure 4 diagrammatically illustrates the procedures that were followed in the implementation of the pilot intervention including decisions that had to be made at the various stages. All participants were reimbursed for transport and all those who were interviewed during or after lunch hour were provided with refreshments.

Data from the 36 pre-coded questionnaires was managed using SPSS version 10 after checking by the investigators for completeness and consistency. The quantitative data was cleaned using the appropriate techniques including double-checking and was analysed using frequencies, percentages, means, standard deviations, cross tabulations, group statistics, matched pair analysis and other statistical tests such as independent sample tests, Chi-Square test and Fischer’s exact test. Figures, percentages and tables were used to summarise the data.

The following measures were taken to remove bias:

•Interviewers were blinded regarding the group the individuals they were interviewing had participated in. The 36 women were randomly assigned to the two interviewers.

•Scoring was done by a different individual before unblinding of groups.

•Individuals involved in presenting the intervention and the placebo programme did not participate in the assessment or in the scoring – scoring was thus independent and blinded.

•The intervention and second assessment were done about eight months after the empirical study. This could have assisted in eliminating the effects of history and maturation of instrument.

The intervention was implemented in accordance with the requirements of the Declaration of Helsinki. Permission was sought in writing and granted by the Principal Investigators at the two sites before data collection for this study. The study was reviewed and approved by two Research Ethics Committees (RECs) at the University of KwaZulu-Natal (Approval number HSS/0679/07D) and the College of Medicine, University of Malawi respectively (Approval P/02/08/612). Written informed consent was sought and obtained from all the study participants after purpose of the intervention as well as the procedures to be followed during the intervention. Those who refused to participate in the intervention for various reasons were excluded (*n*=3). The women who participated in the study were reimbursed for transport using the standard rate applicable at both sites. They were also provided with refreshments in view of the additional time they had to spend at the site.

## Results

### Demographic characteristics

There were 18 women in both the intervention and non-intervention arms of the intervention study who had been randomly assigned. The ages of the 36 women were evenly distributed across the two groups. This distribution was roughly reflective of the distribution in the sample of the empirical study sample (*n*=203). Table 1 shows the age distribution of the intervention sample.

**Table 1:** Distribution of intervention phase participants by age and level of education

**Distribution by age**					
**Age**	**Freq**	**Percent**	**Non intervention arm**	**Intervention arm**	**Cumulative %**
20-25	11	30.6	33.3% (6)	27.8% (5)	30.6
26-30	11	30.6	33.3% (6)	27.8% (5)	61.1
31-35	11	30.6	27.8% (5)	33.3% (6)	91.7
36-40	3	8.3	5.6% (1)	11.1% (2)	100.0
Total	36	100.0	100% (18)	100% (18)	
Distribution of participants by level of education					
Standard 1-4	2	5.6	5.6% (1)	5.6% (1)	5.6
Standard 5-8	24	66.7	72.2 (13)	61.1% (11)	72.2
Form 1-4	10	27.8	22.2% (4)	33.3% (6)	100.0
Total	36	100.0	100 (18)	100% (18)	

The majority of women (24) had 5–8 years of schooling, followed by those who had 1–4 years of secondary education (10). The distribution of the women by years of schooling was evenly balanced across the two groups and consistent with the distribution during the preceding empirical phase of the study, showing the effectiveness of the random sampling method that was adopted. Table 1 also shows the distribution of the intervention sample by years of schooling across the two groups. The majority of the intervention participants had attended up to eight years of primary education.

By the time of implementing the intervention, all microbicide trial participants had already been informed about the microbicide study arm they were in. This was done during the time they were informed about the microbicide study findings. Twenty three (23) of the women who participated in the intervention phase were from either of the two active product arms of the microbicide trial, while 13 were from the trial’s placebo arm. This distribution shows that the random sampling strategy used in selecting participants for the intervention phase led to a balanced sample. The intervention study sample included at least one third from each of the three gel arms of the microbicide study. Eight (8) of the 13 who were on the placebo arm indicated that they felt cheated or betrayed when they were informed about the arm they were on during the study. This finding presented some evidence of false confidence among the participants. It also showed that participants did not appreciate the real possibility of being on placebo or the unproven efficacy of the test product. All eight (8) women reported that they felt betrayed as they believed that they were using an active product which would have protected them.

### Findings on the effectiveness of the intervention

In order to assess the effectiveness of the intervention, the average scores for each concept were compared for the two groups before and after the intervention. From the findings in Table 2 below, it is evident that after the intervention, the mean and median scores improved for both groups on understanding of randomisation, placebo use and personal implications. The fact that both groups experienced increases in scores on understanding of certain concepts suggests the effect of some confounding variables. The termination of the microbicide trial as well as dissemination of the microbicide study results could have served as confounders. The two factors could have reinforced the fact that the microbicide trial was indeed a study and not a programme aimed at HIV prevention. Study nurses reported that upon termination of the microbicide study, they invited the trial participants to bring back to the site any remaining gels, only to find that some of them had shared the gels with their colleagues. The increased scores for the non-intervention arm were however small compared to the increases evidenced by the intervention arm. This finding provides some evidence for the short-term usefulness of the intervention in improving understanding.

**Table 2:** Mean and median scores by group before and after intervention

	**Before intervention (*n=*36)**		**Non intervention group after intervention (*n=*18)**		**Intervention group after intervention (*n=*18)**	
	**Mean**	**Median**	**Mean**	**Median**	**Mean**	**Median**
Randomisation	78	75	78.42	90	92	100
Double-blinding	74.86	78.00	64.83	67	85.17	100
Placebo	56.11	60.00	78.67	83.0	91.61	100
Implications	42.92	44.00	58.17	55	74.11	78
Composite score	60.53	60.00	70.19	67	88.89	100

Before the intervention, there were no differences in terms of the distribution of scores between the intervention group and the non-intervention group as shown in Table 3 below. Table 3 confirms that before the implementation of the intervention, all 36 women were in the low score category. Our results confirm that after the intervention, 13 women in the intervention arm had moved to the high score category of 75% and above as presented in Table 3 below. In the intervention group, after the intervention, no participants remained in the 0-50% score category while five women remained in this category in the non-intervention arm. This finding suggests the effectiveness of the intervention in improving understanding.

**Table 3:** Distribution of composite scores before and after the intervention

**Distribution of scores before the intervention**					
		**Low score 0-49%**	**Low score 50-74%**	**High Score 75+**	**Total**
Non intervention	Frequency	2	16	0	18
	Percentage	11.1%	88.9%	0	100%
Intervention	Frequency	1	17	0	18
	Percentage	5.6%	94.4%	0	100%
TOTAL	Frequency	3	33	0	36
	Percentage	8.3%	91.7%	0	100%
**Distribution of scores after the intervention**					
Non intervention	Frequency	5	13	0	18
	Percentage	27.8%	72.2%	0	100.0%
Intervention	Count		5	13	18
	percentage	0	27.8%	72.2%	100.0%
TOTAL	Frequency	5	18	13	36
	percentage	13.9%	50.0%	36.1%	100.0%

Fischer’s exact test was used to confirm the effect of the intervention on understanding of randomisation. Interestingly, Fischer’s exact test revealed that the influence of the intervention on understanding of randomisation was not statistically significant (*p=0* .075). To assess the effect of the intervention on improving understanding on placebo use, a p*-*value of 0.003 using Fischer’s exact test was obtained. This *p-*value indicated that there was a statistically significant relationship between the intervention and improved understanding of placebo use. Fifteen (15) of the 18 participants in the intervention arm scored higher (75%+) on understanding of placebo use, compared to only 6 on the non-intervention arm. Cross-tabulation of intervention by score on double-blinding also showed that the intervention had a statistically significant effect on scores of understanding of double-blinding. Thirteen women in the intervention arm scored highly (75%+) as compared to only 3 women in the non-intervention arm. A *p-*value of *p=*.001 was obtained using both Pearson’s chi-square and Fischer’s exact test. This *p-*value indicates that there was a statistically significant relationship between the intervention and improved scores on double-blinding. The effect of the intervention on the understanding of personal implications was also assessed. A *p-*value of 0.000 was obtained using Fischer’s exact test and Pearson’s chi square test. This *p*-value confirmed that the intervention had a statistically significant impact on improved understanding of personal implications of research participation. Eleven (11) of the 18 women on the intervention arm scored highly (75%+) on understanding of personal implications in contrast to only one woman on the non-intervention arm.

In an attempt to assess the impact of the intervention on overall understanding of all four areas under study, the independent variable (the intervention) was cross tabulated with the composite score. Table 4 below indicates that 13 of the participants in the intervention arm managed to obtain high scores, while no participant from the non-intervention arm managed to score 75% and above. A *p-*value of 0.0001 was obtained using both Pearson’s chi-square test and Fischer’s exact test, signifying that there was a statistically significant relationship between the intervention and the improved composite scores obtained.

**Table 4:** Relationship between intervention and composite score

		**Low scorer 0-74%**	**High scorer 75%+**	**Total**
Non interv.	Frequency	18	0	18
	Percentage	100.0%	0	100.0%
Intervention	frequency	5	13	18
	Percentage	27.8%	72.2%	100.0%
TOTAL	Total Count	23	13	36
	Total %	63.9%	36.1%	100.0%
				*P=*0.0001

Matched pair analysis of the scores of the participants in the intervention arm before and after the intervention indicates that all participants experienced some significant gains in scores except for one who experienced a drop of 2% from 65% before the intervention to 63% after the intervention. Nine of 18 respondents showed very large gains of 20% and above, while 8 of the 18 respondents experienced some gains of between 10% and 19%. Only one participant experienced a minimal loss of 2% (Refer to Table 5).

**Table 5:** **Matched pair analysis of composite scores for Intervention group before and after intervention for intervention group (*n=*18)**

**Participant number**	**Composite score after intervention**	**Composite Score before interv.**	**Difference**	**Negative/Positive Gain**	**Magnitude of gain**
164	63	50	+13	**Pos**	Significant
093	70	65	+15	**Pos**	Significant
112	70	58	+12	**Pos**	Significant
150	70	58	+12	**Pos**	Significant
183	85	70	+12	**Pos**	Significant
136	89	68	+13	**Pos**	Significant
158	89	63	+13	**Pos**	Significant
184	89	60	+29	**Pos**	Very sig
191	89	60	+29	**Pos**	Very sig
179	93	68	+25	**Pos**	Very sig
083	96	70	+26	**Pos**	Very sig
157	96	58	+38	**Pos**	Very sig
198	96	40	+56	**Pos**	Very sig
063	100	58	+42	**Pos**	Very sig
202	100	50	+50	**Pos**	Very sig
096	81	68	+13	**Pos**	Significant
135	85	63	+22	**Pos**	Very Sig
070	63	65	−2	Neg	Minimal

Matched pair analysis of the scores of the participants from the non-intervention group before and after the intervention revealed that the majority of the participants scored lower during the second assessment. Twelve of the 18 respondents experienced negative gains ranging from −1 to as high as −28, and two respondents did not experience any gain in their scores. This finding confirms the suggestion that history and maturation of instrument had very minimal bias on the scores since the second assessment was done about 8 months after the first assessment. The difference between the gains of the intervention arms and those of the non-intervention arm also confirms that the standard package that was used for the non-intervention did not influence the scores of the non-intervention arm in a significant way. Interestingly, seven (7) of the 18 non-intervention respondents experienced losses of 10% and above. Such losses were classified as significant losses (10-19%) and very significant (20% and above). Only two respondents experienced significant gains of between 10-19% (Refer to Table 6).

**Table 6:** **Matched pair analysis of composite scores for Non Intervention group before and after intervention (*n=*18)**

**Participant number**	**Composite score after inter**	**Score before intervention**	**Net gain in scores**	**Negative/Positive gain**	**Magnitude of gain/loss**
163	33	53	−20	***Neg***	Very significant
139	40	58	−18	***Neg***	Significant
116	44	48	−4	***Neg***	Minimal
089	55	70	−15	***Neg***	Significant
120	55	55	0	***Zero***	Nil
134	55	55	0	***Zero***	Nil
155	55	40	+15	Pos	Significant
168	55	53	+2	Pos	Minimal
105	59	60	−1	***Neg***	Minimal
085	63	68	−5	***Neg***	Minimal
181	63	73	−10	***Neg***	Significant
100	67	65	+2	Pos	Minimal
115	67	68	−1	***Neg***	Minimal
201	67	53	+12	Pos	Significant
094	70	65	+5	Pos	Minimal
124	48	73	−28	***Neg***	Very Significant
101	63	70	−7	***Neg***	Minimal
194	44	60	−14	***Neg***	Significant

Group statistics and independent sample tests were also calculated for the two groups. Table 7 below show the group statistics on all the categories that were scored after the intervention. Table 7 also shows that the mean scores obtained by members from both groups before the intervention were almost similar. After the intervention, the mean and median values for the intervention group were higher than those obtained by the non-intervention group for all areas under study.

**Table 7:** Intervention and non-intervention Group Statistics before and after the intervention

	**Intervention/non intervention**	**N**	**Mean before**	**Mean after**	**Std dev before**	**Std dev after**	**Std error mean before**	**Std error mean after**
Composite score	Non intervention	18	60.39	55.72	9.26	10.45	2.18	2.46
	Intervention	18	60.67	92.22	7.93	15.55	1.87	3.67
Implications	Non intervention	18	41.78	42.22	9.99	13.36	2.36	3.15
	Intervention	18	44.06	84.67	16.40	12.36	3.87	2.91
Double-blinding	Non intervention	18	54.44	44.50	20.36	34.14	4.80	8.05
	Intervention	18	57.78	74.11	22.64	19.60	5.34	4.62
Placebo use	Non intervention	18	74.83	65.72	20.43	21.81	4.81	5.14
	Intervention	18	74.89	85.17	15.42	26.22	3.63	6.18
Randomisation	Non intervention	18	79.33	64.61	12.19	29.17	2.87	6.88
	Intervention	18	76.67	91.61	13.56	13.14	3.20	3.10

Independent sample tests were done before and after the intervention to check on the usefulness of the intervention at group level. *P-*values above 0.005 were obtained for scores before the intervention, and *p-*values below 0.005 were obtained for all the four scores after the intervention. The *p*-values before the intervention show that, before the intervention, there were no significant differences between the two groups while those after the intervention confirm that there were significant differences between the groups. This finding is particularly important as it shows that the samples were independent and that the intervention had a positive effect on understanding of all four areas under consideration in this study.

Several interesting observations arose during the implementation of the intervention. The intervention was presented in an environment that encouraged discussion and the participants were free to interrupt the presenter and make comments or seek clarification. Of interest were some of the comments that came from participants during and after the intervention. The following comments specifically shed more light on the usefulness of the intervention in improving understanding:

"So the farmers who participated in the experiment are supposed to buy the new fertilizer at a reduced price. If the company was selling the fertilizer at K100, they should sell it to the farmers at K50 because the farmers would have assisted in the development of the fertilizer."

"The fifty farmers who got the test product benefitted immensely through the improved yields. The company was supposed to give the new fertiliser to the other fifty so that they can benefit as well since they participated in the research."

The above comments clearly show that these particular women had clearly realised that the farmers in Ntcheu had been used as part of a study and the aim of the study was the generation of new information that could be used in establishing if the new fertilizer was effective. The issue of justice and benefit sharing by giving the test product to the control arm in the event of successful results has been an area of active debate in research ethics recently. It was interesting that two of the participants could justify claim to benefits in those ways. The participants had clearly realised that, by participating in the research, they were going to assist others in future, just as the farmers participated in the research which eventually led to improvements in yields for the whole nation.

The majority of the women were grateful to the presenter for having presented the information in a way which was easy to understand.

"***LLP7***: On behalf of my friends, I am grateful to you for your coming. To be frank we have learnt a lot from this discussion about the Microbicide study. We have been participating in the microbicide study. The way you explained it, was as if you were talking about a new study. Your questions made us think about what we went through during our participation in this study and it will help us to remember this study forever. So, we don’t take our participation in the discussion for granted- we thank you so much!"

The majority of women were very appreciative of the microbicide study as they indicated that it had assisted them in learning about their HIV status, and they were assisted by the staff whenever they had health problems.

## Discussion

The findings of this pilot intervention, which aimed at testing the effectiveness of an intervention aimed at improving understanding of the three concepts (randomisation, double-blinding, placebo use) and their personal implications, suggest that the intervention was effective in improving understanding. The findings suggest that understanding of trial concepts can be improved if explained in clear and local terms. Women in both the intervention and non-intervention arms were selected because they had obtained low scores in the initial test that was administered during the empirical study that we conducted on trial participants understanding prior to development and implementation of this intervention (under review). After the intervention, the majority of women on the intervention arm scored significantly higher on the understanding of all four areas under study (randomisation, double-blinding, placebo use and their personal implications) compared to those in the non-intervention arm.

The success of the intervention can be attributed to various factors. Studies on previous intervention were reviewed and points taken on weaknesses, strengths and areas of improvement. All these were taken into consideration in the development of the intervention which was tested in this study. The intervention was delivered in ChiChewa, which is the dominant language in Malawi. The use of local language in informed consent is emphasised in earlier works and was aimed at ensuring that the information disclosed would be meaningful to all respondents, regardless of their educational level [24,40]. Throughout the intervention, layman’s language was used in explaining the procedures and their justifications. As suggested in an earlier work, examples were taken from agriculture since Malawi is an agriculture-based country and every individual is conversant with agriculture [35]. Adequate time was provided for the implementation of the intervention. In this case, the aim was only to improve understanding, unlike in many real clinical trial settings where staff may be preoccupied with meeting accrual targets rather than enhancing understanding.

A hierarchical and modular approach to providing information was used. As recommended in literature, care was taken to ensure that information was provided on each concept to cover all three areas, namely procedures, purpose, and personal implications [16]. The procedures were explained in the form of a story which was interesting and easy to follow and relate to as suggested by findings from a previous study [35]. In Malawian culture, as has been observed to be the case in the majority of African cultures, folktales are used as a useful way of passing important lessons to individuals [32]. Materials were presented in a way which was easy to understand – a PowerPoint presentation, which included colourful pictures, was designed and used as a way of supplementing the information about research, the microbicide study and the procedures under study, as well as their implications. This study corroborates previous studies that have reported that participants can find information meaningful irrespective of their educational level [12,24,40,41]. Inclusion of purpose, justification and implications of the research procedures and research participation was specifically important. The intervention not only focused on the three concepts but brought the three concepts into context so as to make the information more meaningful. Informed consent procedures are often tailored towards mentioning procedures without necessarily describing the procedures in meaningful ways [4].

During the intervention, participants who had understood the concepts were asked to repeat what they had understood to the presenter or to their colleagues. Participants were also allowed to discuss among themselves during and after the intervention. These strategies have been reported to be useful in improving understanding [35,37].The intervention was presented by a staff member from the Centre for Bioethics at the College of Medicine. The presenter was not a member of the microbicide trial research team at the site. During the implementation of the intervention, the microbicide trial staffs were not invited. This ensured that the women would discuss issues freely during the intervention. The presenter was familiar with the microbicide project and had a lot of experience in clinical trial issues, including key methodologic aspects of research. Research staff knowledge and experience with research has been cited as playing an important role in improving informed consent [38]. The findings on the effectiveness of the intervention are consistent with other findings which showed that story-based interventions were useful as they facilitated understanding of concepts and procedures [21,23]. The effect of the intervention on recruitment and retention was not evaluated since the microbicide study had already come to an end. It would have been interesting to test the suggestion by others that provision of adequate information may actually lead to greater understanding, which may ultimately lead to lower enrolment rates through more ‘informed refusals’ [42].

During the planning of the intervention, it had been planned and agreed that the intervention would be introduced to the 18 women in the non-intervention arm if it had been found to be acceptable. This plan was obviously overtaken by events as the intervention was implemented well after the microbicide trial had been concluded. The reason why it had been decided that the intervention would be disseminated to the women in the non-intervention arm in the first place was aimed at ensuring that those women also benefitted directly from the intervention. Tracking of the women for the intervention phase had also proven difficult. It was therefore agreed that the findings from this study would be disseminated at the two sites so that the principal investigators could utilise the findings in other and future trials, if they wished. The dissemination of the intervention to the 18 women in the non-intervention arm would not however not serve a critical purpose because the microbicide trial had already closed. More importantly however, the 18 women in the non-intervention arm benefited from a session on cervical cancer that was conducted as part of the non-intervention package. Plans are in place for the researcher to disseminate the findings at the two sites in Malawi as well as to conduct a larger study. The dissemination of findings on the usefulness of the intervention to the 36 women would have been a good opportunity to document their views on the acceptability and challenges related to the intervention.

It is important to highlight the possibility of some confounding biases. Time is an important factor in any study which looks at a particular phenomenon over time. With time, individuals learn more about research and about the products being studied. With more history and exposure, it means they are in a better position to give the correct answers when asked questions on the subject. The two instruments used in the main study and intervention study covered similar questions. It is therefore possible that the bias of maturation could have been real. It is however important to note that there was a time lag of more than 8 months between the first assessment and the second assessment. This time lag could have weakened the bias caused by instrument maturity. In any case the maturation would have affected the intervention and non-intervention groups equally.

Results from the microbicide study were disseminated to all participants around March 2009. The first assessment had been conducted between September and November 2008. The intervention was introduced and evaluated in July 2009, more than three months after the dissemination of the microbicide study findings. It is therefore possible that the dissemination of findings could have significantly affected the impact of the intervention on understanding. It is important to note, however, that this would have applied equally to both the intervention arm and the non-intervention arm. The women in the two groups were all familiar with the research procedures under study. It can be concluded that the differences between the intervention and non-intervention arms were attributable to the intervention.

## Conclusions

An intervention developed and implemented as part of this study showed positive findings. Evaluation of the intervention suggests that it was useful in improving understanding of the key concepts (randomisation, double-blinding, placebo use, and personal implications of these) under study. More importantly, the study has shown that, if information on scientific procedures is provided in a meaningful, structured, locally relevant and complete way, it is possible to facilitate adequate understanding. The intervention which was tested through this study may guide future researchers in implementing more effective measures to maximise participants’ understanding of essential clinical trial procedures. More importantly, the evidence from the intervention is encouraging as it serves as empirical proof that understanding can be improved if researchers use accessible language and examples that demystify research, and present research as a process which is aimed at improving health care decisions and tools. While the intervention was tested among real trial participants, it is possible that there were several confounding variables that could not be controlled. There is however need to further test this intervention in an active clinical trial setting using participants that are in the process of considering participation in a real trial in order to test impact on both short-term recall and long-term memory. Testing the intervention using participants that are considering trial participation may also assist in answering the question whether improving understanding may affect willingness to participate in a trial.

## Competing interests

PN is on the Board of Editorial Advisors of BMC Medical Ethics. None of the authors have an apparent COI.

## Authors’ contributions

PN contributed in various ways in the work reported in this paper; serving as PI, conception, methodology, data collection, data analysis, initial draft, editing and approval of final version. DW contributed in various ways in the work reported in this paper including; supervision, refinement of research problem, methodology, study tools; guidance in data analysis; inputs to various versions of manuscript, editing and approval of final version. ENM contributed in various ways in the work reported in this paper including; refinement of research problem and methodology, development of tools, data analysis, inputs to various versions of manuscript, editing and approval of final version. FM contributed in various ways in the work reported in this paper including; refinement of methodology, data collection, data analysis, inputs to various versions of manuscript, editing and approval of final version. All authors read and approved the final manuscript.

## Pre-publication history

The pre-publication history for this paper can be accessed here:

http://www.biomedcentral.com/1472-6939/13/29/prepub
